# Understanding the Development of Minimum Unit Pricing of Alcohol in Scotland: A Qualitative Study of the Policy Process

**DOI:** 10.1371/journal.pone.0091185

**Published:** 2014-03-26

**Authors:** Srinivasa Vittal Katikireddi, Shona Hilton, Chris Bonell, Lyndal Bond

**Affiliations:** 1 MRC/CSO Social and Public Health Sciences Unit, University of Glasgow, Glasgow, United Kingdom; 2 Department of Public Health and Policy, NHS Lothian, Edinburgh, United Kingdom; 3 Department of Social Policy and Intervention, University of Oxford, Oxford, United Kingdom; 4 Centre of Excellence in Intervention and Prevention Science, Melbourne, Australia; Edinburgh University, United Kingdom

## Abstract

**Background:**

Minimum unit pricing of alcohol is a novel public health policy with the potential to improve population health and reduce health inequalities. Theories of the policy process may help to understand the development of policy innovation and in turn identify lessons for future public health research and practice. This study aims to explain minimum unit pricing’s development by taking a ‘multiple-lenses’ approach to understanding the policy process. In particular, we apply three perspectives of the policy process (Kingdon’s multiple streams, Punctuated-Equilibrium Theory, Multi-Level Governance) to understand how and why minimum unit pricing has developed in Scotland and describe implications for efforts to develop evidence-informed policymaking.

**Methods:**

Semi-structured interviews were conducted with policy actors (politicians, civil servants, academics, advocates, industry representatives) involved in the development of MUP (n = 36). Interviewees were asked about the policy process and the role of evidence in policy development. Data from two other sources (a review of policy documents and an analysis of evidence submission documents to the Scottish Parliament) were used for triangulation.

**Findings:**

The three perspectives provide complementary understandings of the policy process. Evidence has played an important role in presenting the policy issue of alcohol as a problem requiring action. Scotland-specific data and a change in the policy ‘image’ to a population-based problem contributed to making alcohol-related harms a priority for action. The limited powers of Scottish Government help explain the type of price intervention pursued while distinct aspects of the Scottish political climate favoured the pursuit of price-based interventions.

**Conclusions:**

Evidence has played a crucial but complex role in the development of an innovative policy. Utilising different political science theories helps explain different aspects of the policy process, with Multi-Level Governance particularly useful for highlighting important lessons for the future of public health policy.

## Introduction

Minimum unit pricing (MUP) of alcohol is a novel public health policy that seeks to reduce the adverse public health consequences of alcohol consumption [1], [2]. It has also been identified as a potential measure for reducing health inequalities since alcohol-related harms are strongly socially patterned [3]. Alcohol taxation has long been used as a method to address health and social concerns as well as raise government revenue [4]. Other forms of floor pricing that prevent the sale of very cheap alcohol have been successfully elsewhere, with the most comparable being reference pricing that has been introduced in some Canadian provinces [5], [6]. MUP builds on these initiatives by linking the lowest price paid for alcohol to its content of pure alcohol, defined on the basis of ‘units’, where one unit equals 8g of ethanol [7]. The policy was first introduced into legislation in Scotland in May 2012 but its implementation has been delayed as a result of industry-initiated legal challenges [8], [9].

Despite the existence of somewhat similar policies internationally, MUP represents an important development in alcohol policy and differs from the Canadian policy experience in a number of ways. First, the nature of the policy differs. Rather than the introduction of a variable floor price, which may encourage switching in consumption from one product to another to maintain alcohol intake, MUP introduces a price threshold that is uniform across all alcohol products [10], [11]. Second, MUP is being introduced into a competitive market environment, rather than a government-controlled monopoly. Third, the policy has been pursued for public health purposes, rather than primarily for revenue-raising reasons [12].

Within the health field, there has been increasing interest in improving the use of research findings in policymaking [13]. However, there are continuing indications of the limited impact of evidence in public health policy [14], [15]. In this article, we aim to explain the development of MUP in Scotland by drawing on theories of the policy process and identify lessons for evidence-informed policymaking. In the next section, we provide a brief summary of three theories – Kingdon’s Multiple Streams model; Punctuated-Equilibrium Theory and Multi-Level Governance – which informed our analysis. After describing our methods, a descriptive overview of the policy process forms the first part of the results. We then used a ‘multiple-lenses’ approach to understand MUP’s development, considering each of the three theories in turn. Finally, we outline a number of lessons for the movement towards evidence- informed public health and reflect on the limitations of the study.

### Theories of the Policy Process

A wide variety of theories now exist to explain the policy process but none appear satisfactory for all purposes [16], [17], [18]. Historically, policy was assumed to occur in a ‘rational’ manner which involved passage through a number of distinct stages which together constitute a ‘policy cycle’ [19], [20]. While this model continues to underpin (often implicitly) the perspective of many health researchers and those involved in policy [21], it is not supported by empirical evidence and does not recognise the constraints on rational decision-making or the role of implementers in policy as it is enacted [17], [19]. Despite these limitations, the stages model presents a helpful heuristic device and many of the processes described above often do occur during the policy process, albeit in a non-linear manner.

The limitations of the stages heuristic resulted in the development of alternative models – three of which we draw upon in our analysis. Kingdon’s Multiple Streams model seeks to understand what factors explain the process by which the multitude of policy issues that could be considered by policymakers, results in the relatively few policy problems that are actually considered in detail [22]. He identified three factors, referred to as ‘streams’, which must come together for a policy to develop. The existence of a policy issue that is construed as worthy of policy intervention constitutes the *problem* stream. The *policy* stream refers to the availability of a solution that is viewed as having the potential to address the problem. The *politics* stream refers to the political climate operating which may either help or hinder the consideration of a specific policy issue (for example, including the political parties dominant at the time). These three streams can be ‘coupled’ by ‘policy entrepreneurs’; advocates of a proposed policy solution who are skilful communicators and are willing to sustain their efforts for prolonged periods of time.

The second theory, Punctuated-Equilibrium Theory, seeks to explain the observation that many areas of public policy exhibited little policy change (i.e. are in ‘equilibrium’) while a few areas experience rapid shifts in policy (‘punctuations’) [23]. It argues that the time constraints policymakers operate under result in them being unable to focus on all areas of public policy simultaneously. High-level policymakers therefore leave most areas to communities of experts while they focus on the areas that have become ‘hot topics’, attracting the attention of the media and a broader group of actors than previously engaged. Policy areas that are undergoing punctuations therefore experience an increased tendency for radical policy change as a result. In keeping with Kingdon’s Multiple Streams Model, Punctuated-Equilibrium Theory suggests that a change in the ‘framing’ (or perception) of a policy issue or a well-respected indicator can be crucial in triggering increasing interest in an existing policy problem.

The third perspective that informs this paper is Multi-Level Governance. In contrast to the other two theories, Multi-Level Governance does not provide a theory of the policy process as such but rather draws analytical attention to ongoing changes in institutional responsibilities, derived from experiences in Europe [24], [25], [26]. A multi-level perspective theorises a shift in power from one central governmental state authority (such as the UK Government) to a range of institutions that operate both above (for example, the European Union) and below the nation state (e.g. the devolved Scottish Parliament). It also theorises an increase in the involvement of non-governmental and private sector actors, signalling a move from ‘government’ to ‘governance’. Reflecting this academic perspective has been a growing interest in the impact of devolution – the increase of governmental institutions operating below the state as illustrated in the UK by the Welsh Assembly and the Scottish Government [27].

## Materials and Methods

### Ethics Statement

Ethical approval was obtained from the University of Glasgow’s College of Medicine and Veterinary Science research ethics committee. Informed consent was sought from each participant in a process approved by the ethics committee and documented in a written consent form.

A qualitative case study approach [28] has been adopted to investigate MUP’s development. We drew on two main sources of data: semi-structured interviews with policy actors and a review of relevant publicly available policy documents. Potential interviewees were mainly identified from the document analysis and usually initially approached by e-mail to request their participation in a research study on the relationship between evidence and alcohol policy. Of those contacted, seven potential participants were unwilling to participate (usually for reasons of lack of time) and interviews could not be scheduled with a further five. Thirty-six interviews were conducted by SVK (a public health doctor) with purposively selected policy actors (5 politicians, 10 civil servants, 7 advocates for MUP, 6 industry-related actors and 8 academics) directly involved in MUP debates in Scotland and/or the UK. In addition to sampling participants on the basis of the above five sectors, diversity was sought within each sector (political party membership for politicians, nature of industry-related actor and area of focus for advocacy groups) and in relation to supportiveness or hostility to MUP. Interviews typically lasted 45–60 minutes and were conducted either face-to-face (usually at the participant’s place of work) or over the telephone. Recruitment continued until adequate diversity was obtained in the sample and no major new themes emerged in the data [29]. Contemporaneous handwritten fieldwork notes were kept during the data collection and analysis process.

Given the small nature of the policy community we were seeking to access, we sought separate written consent for interview participation, audio-recording the interview, attribution to the interviewee’s broad sector (for example, civil servant, politician etc.) and the use of quotations [30]. All but four interviewees agreed to full participation. Following the interview, transcripts were amended by noting sections not for quotation and making limited changes to the text to reduce the risks of disclosure in consultation with interviewees. Finally, interviewees were given the opportunity to review a copy of the transcript and make limited suggestions for further amendments to ensure confidentiality.

Interview data were read repeatedly and thematic content analysis conducted by SVK [31]. Coding initially proceeded inductively with descriptive codes being used to organise the data with the assistance of NVivo 9. Following this, data relevant to specific political science theories were coded using sets of ‘tree’ codes for each political science theory and subcodes used to capture a key aspect of each theory. New codes were used to capture further inductive themes in the data and a broader set of theories (for example, theories relating to policy transfer and ideas [32], [33], [34]) were also during the coding process. A relatively parsimonious account of the policy’s development, and in particular one which helped explain the origins of MUP, was then sought through an iterative process of analysis. The principle of the constant-comparative method was used to help identify explanations for patterns within the data, while also paying appropriate attention to contradictions and tensions within the data.

The review of publicly available documents sought to include published documents, which reported on quantification of alcohol-related issues (either positively or negatively), the causes and/or consequences of alcohol use, identification or debate over proposed action(s) and advocacy for specific actions. The search was conducted online and by contacting the Scottish and UK governments, health service organisations and interest groups (including health actors, other civil society organisations and a wide variety of industry groups). Interest groups were identified by reviewing the actors responding to public consultations to MUP debates within Scotland [35] and the UK [36].

Documents submitted by policy stakeholders in response to the Scottish Parliament’s Health and Sport Committee consultation (conducted from November 2009 to January 2010) were retrieved from the Scottish Parliament website and systematically analysed using thematic content analysis to understand the framing of arguments presented by policy actors. Further details of the methodology are described elsewhere [37].

## Results and Discussion

### The Development of the Minimum Unit Pricing Policy in Scotland

Scotland is one of the four constituent countries of the United Kingdom and political decisions at the central government-level have been made at the Westminster Parliament in London since 1707 [38]. Throughout this time, Scotland maintained separate legal (including alcohol licensing) and education systems. In 1999, a separate Scottish Parliament was established with responsibility over some ‘devolved’ policy areas (such as health, education and social care) but with other areas ‘reserved’ to Westminster (including trade and foreign affairs). Devolution has allowed Scotland the freedom to pursue policy differently from England in some areas. For example, Scotland was the first country within the UK to pass legislation to ban smoking in public places [39]. The first two Scottish Parliamentary elections resulted in coalition governments between the Labour and Liberal Democrat Parties. The Scottish National Party (SNP) has been in government since 2007, initially with a minority but gaining a majority in 2011. Key events are highlighted in the timeline (see Table 1).

**Table 1:** Timeline of Milestones in the Development of Minimum Unit Pricing in Scotland.

Date	Event
May 1999	First Scottish Parliamentary elections
Jan 2002	First Scottish Alcohol Strategy, the ‘Plan for Action on Alcohol Problems’ published
March 2004	‘Alcohol Harm Reduction Strategy for England’ published
March 2006	Scotland passes ban on smoking in public places
Feb 2007	Update on first Scottish Alcohol Strategy
May 2007	SNP elected as minority Scottish Government
July 2007	England passes ban on smoking in public places
Sep 2007	SHAAP Expert workshop results in first public report advocating minimum unit pricing
Oct 2007	Justice Minister, Kenny MacAskill, argues in Scottish Parliament that regulation to address low-cost alcohol is necessary
June 2008	Discussion paper ‘Changing Scotland’s Relationship with Alcohol’ published
Nov 2008	SHAAP-commissioned econometric modelling short report published
Dec 2008	First set of systematic reviews and econometric modelling studies commissioned by the UK Department of Health and published by Sheffield University
Mar 2009	Departmental responsibility for addressing alcohol-related harms transferred from Justice to Health
May 2009	Scottish Government’s post-consultation ‘Framework for Action’ published
Sep 2009	First Scottish version of the Sheffield econometric models published
Nov 2009	Alcohol etc (Scotland) Bill first introduced to the Scottish Parliament including provisions to introduce minimum unit pricing
Nov 2010	Alcohol etc (Scotland) Bill passed without minimum unit pricing
May 2011	SNP elected as a majority Scottish Government
Mar 2012	UK Government announces plans to introduce minimum unit pricing in England in the second ‘Government’s Alcohol Strategy’
May 2012	Alcohol (Minimum Pricing) (Scotland) Bill passed by the Scottish Parliament
July 2012	Legal challenges to the introduction of minimum unit pricing in Scotland made by the Scotch Whisky Association
Nov 2012	UK Government announces its intention to introduce MUP at a 45 pence per unit level for England and Wales

### Alcohol Harms in Scotland

There have been longstanding concerns about alcohol consumption and harmful drinking patterns in Scotland and the UK [40]. While many professionals perceived an increase in alcohol harms since the 1990s, there was initially a lack of epidemiological data to quantify the problem. Scotland introduced its first post-devolution alcohol strategy in January 2002 [41], two years before England. The ‘Plan for Action on Alcohol Problems’ (the Alcohol Plan) was the first report to bring together data on the alcohol market, alcohol consumption, social harms and health harms and demonstrated the considerable overall alcohol burden [41]. The Plan was introduced by a Labour-Liberal Democrat coalition following a commissioned review of effective and cost-effective measures to reduce alcohol harms [42]. It set out a broad range of measures to reduce alcohol-related harms including education, provision of services and licensing reform but maintained an emphasis on addressing problem drinkers and individual responsibility [41]. This framing of policy as a matter of addressing the minority of drinkers was reflected in two specific priority areas: addressing binge drinking and reducing drinking by children and young people [43].

A key component of the Alcohol Plan was the introduction of the Licensing (Scotland) Act 2005 which was fully enacted in September 2009 [44], [45]. As well as simplifying the licensing regime, this introduced five licensing objectives, including ‘the protection and improvement of public health’. This objective was seen as an important step forward in making public health considerations central to alcohol licensing. The Act also banned promotions encouraging excessive consumption in on-sales premises, such as ‘happy hours’ (when alcohol products are discounted for a time-limited period during the day). At the time of the Act’s drafting, there was recognition that further action might be required to address off-sales [43].

### The Importance of Price for Alcohol-Related Harms

One important cause of alcohol-related harms that has been increasingly focused on is the relationship between increasing alcohol affordability and levels of consumption [46]. This relationship has been widely accepted with considerable supportive evidence existing [47], [48]. From a UK perspective, while alcohol prices have increased with inflation, increased living standards had made alcohol 66% more affordable in 2009 than in 1987, with a growing price differential between off-sales and on-sales prices [49]. Supermarkets have contributed to this increasing affordability by engaging in aggressive cost-cutting of alcohol [50], sometimes selling alcohol as a loss leader and/or below the cost of duty alone [51], [52]. Paralleling the increased affordability within off-sales, there has been a shift in consumption from the licensed trade to off-licenses (and particularly supermarkets) [53], [54]. It has been argued that this growing price disparity has led to increased consumption at home, including prior to leaving for a night out [55].

Towards the end of the Labour-Liberal Democrat coalition (1999–2007), a paper in the Lancet investigated trends in alcohol-related harms by analysing liver cirrhosis deaths and found a more than doubling in death rates in Scottish men from 1987–1991 to 1997–2001 [56]. This increase greatly exceeded changes in England and ran counter to trends across most of Western Europe, adding impetus to calls for further action.

Within Scotland, the importance of price as a key mechanism to tackle alcohol-related harms was highlighted by two Scottish organisations. The national agency for health improvement, NHS Health Scotland, was asked by the Labour-Liberal Democrat administration to review the alcohol evidence base to develop a logic model to inform a new Scottish Government strategy to address alcohol harms [57]. To develop the logic model, the agency reviewed research evidence but also considered plausible theory and existing policy [58]. This logic model highlighted the central importance of the “Reduction in individual ***and population consumption*** [emphasis added]” and the need to tackle alcohol affordability.

Price was also highlighted in the run-up to the 2007 election campaign by a new Scottish public health advocacy group: Scottish Health Action on Alcohol Problems (SHAAP), established by the Scottish Medical Royal Colleges and Faculties in response to concern about increasing alcohol-related harms [59]. The organisation was funded by Scottish Government with a commitment that it could operate entirely independently and a remit “to raise awareness about alcohol-related harm and to promote solutions based on the best available evidence.” [60].

### A Move to a Population-based Approach

In May 2007, the incumbent Labour-led administration was replaced by a Scottish National Party (SNP) minority government. Until this time, the Labour Party was also in power in the UK Westminster Government and the presence of the same party within both governments may have served to curtail potential policy divergence. While both the Labour and SNP parties highlighted alcohol-related health harms in their election manifestos, their approaches to tackling the problem differed. In 2007, the Scottish Labour Party manifesto emphasised the importance of its “ground-breaking partnerships” with alcohol-related industries while also noting the importance of individual responsibility. The SNP manifesto placed less emphasis on individual responsibility but also advocated a broad partnership approach (including with alcohol industries). However, an important difference was the inclusion of price-based measures, noting it “is not acceptable that a bottle of water can be more expensive than alcohol” [61].

### The Development of MUP in Scotland

In September 2007, SHAAP convened an expert group to investigate the relationship between alcohol price and harms. Its report built on the international evidence base [4], emphasising the need for a ‘whole-population approach’ to address alcohol-related harms [60]. The report described the epidemiology of alcohol harms in Scotland and noted price as a key driver. Recommendations to the Scottish Government from the expert workshop included the introduction of a floor pricing mechanism (with MUP being specifically discussed within the workshop), extending the ban on irresponsible alcohol promotions from on-licenses to off-licenses, advocating to the UK Westminster Government for increased alcohol taxation and for this to be linked to alcohol strength. Subsequently, SHAAP strongly advocated for MUP by regularly engaging with politicians, producing policy briefings and pro-actively communicating with mass media.

In parallel to the advocacy instigated by SHAAP, a team at Sheffield University was asked to carry out a review of the evidence base on alcohol pricing and promotion for the UK Government [47]. The Sheffield team carried out an econometric modelling exercise to compare different pricing interventions including duty increases, MUP (for a range of levels –20 pence per unit at the lowest level to 70 pence per unit at the highest) and restrictions on off-trade price promotions [11]. Importantly, this model indicated MUP most affected those at highest risk of harms when compared to taxation-based measures.

Similar efforts to use econometric modelling to estimate the impacts of MUP were pursued within Scotland. The first Scottish report, commissioned by SHAAP, provided estimates of changes in price and consumption, and also examined the potential impact on household expenditure by differing levels of income (given concerns about impacts on lower-income subgroups) [62]. Following the formal adoption of MUP in official Scottish Government policy in May 2009 [2], the Sheffield team was asked to produce another version of their model using Scottish data. This was first published in September 2009 [63] and subsequently updated twice. These peer-reviewed Sheffield models were not accepted by all MSPs, who drew on a critique of MUP funded by the brewer SABMiller [64].

### Changing Scotland’s Relationship with Alcohol: The Second Scottish Alcohol Strategy

Following the SNP’s election, the Justice Minister, Kenny MacAskill, presented tackling cheap alcohol as a priority within Scottish Parliamentary debates. Initially, the Scottish Government intended to publish an alcohol strategy within the first few months of its administration. However, it realised a radical strategy was necessary and therefore published the discussion paper, ‘Changing Scotland’s Relationship with Alcohol’, in June 2008 instead and consulted on a number of proposals including MUP [65]. The paper explicitly made links to overarching government aims to deliver a “Wealthier and Fairer, Safer and Stronger, Healthier and Smarter Scotland”. Importantly, the approach moved from addressing problem drinkers to addressing population consumption [65]. This innovative ‘whole-population approach’ is reflected by one of the four key areas for action being to reduce overall alcohol consumption. In addition to MUP, a number of other controversial interventions were consulted on including: raising the minimum purchase age to 21 in off-sales; separate checkouts for alcohol sales; and introducing a ‘social responsibility fee’ for some alcohol retailers. Considerable Scottish Parliamentary and media debate occurred following the discussion paper, with the formal consultation receiving 259 responses from individuals and 207 responses from organisations [66].

### Scottish Parliamentary Considerations of MUP

The passage of MUP into legislation in Scotland has not been straightforward. There has been a prolonged and heated political debate as to whether this legislative measure was the most ‘appropriate’ means of tackling alcohol-related harms in Scotland.

On the first attempt to introduce MUP, only the SNP (as a minority government) was openly supportive, with the other major political parties opposed. However, several of the measures included in the Bill (such as a ban on off-trade promotions) had broad support. Despite repeated attempts by the minority SNP Government to keep the MUP component of the Bill, this measure was ultimately withdrawn and the Bill passed without the MUP provision in November 2010.

In May 2011, Scottish Parliamentary elections resulted in the SNP (which had included MUP in its manifesto [67]) gaining an overall majority of seats in the Scottish Parliament [68]. A second MUP Bill was introduced and while the SNP majority government no longer required the support of opposition parties to pass legislation introducing MUP, two of the three opposition parties supported the Bill with the inclusion of a ‘sunset clause’ [69]. A change in leadership within the Liberal Democrats facilitated their change of position while the Conservative-led UK Government had started considering MUP themselves. During the latter stages of MUP’s second consideration for legislation, evidence related to reference pricing from Canada helped provide confirmation of public health benefits following the introduction of a comparable minimum price scheme [5], [6]. On 24^th^ May 2012, the Alcohol (Minimum Pricing) (Scotland) Bill was passed with 86 voting in favour of the measure, one vote against and 32 abstaining [70].

The Scottish Government initially planned to implement MUP in April 2013 but has been delayed after the Scotch Whisky Association followed through on its pre-legislation threats and challenged the legality of MUP on the grounds that it could distort trade within the European Union and questioned the Scottish Government’s legal competence to introduce the measure [71]. This legal challenge has been quashed but policy implementation will be delayed following an appeal [9].

### Explaining the MUP Policy Process

To understand the process by which MUP developed, each of the previously described theories of the policy process will be applied. Rather than trying to identify an optimal theory, insights will be sought from each of the theories through a ‘multiple-lenses’ approach [72].

#### Kingdon’s Multiple Streams

According to the Multiple Streams model of the policy process, three streams would be expected to coalesce to bring about MUP policy [73]. In the ‘problem’ stream, a change in a well-respected indicator could help highlight a problem for policymakers. Epidemiological data that described the burden of alcohol-related harms appeared particularly influential. The Lancet study [56] demonstrating the large burden and adverse trend in Scotland was seen as particularly influential:

*Civil Servant: […] there is no doubt that the single most compelling graph that we showed ministers was that taken from a paper published in the Lancet by David Leon and Jim McCambridge which shows deaths from liver cirrhosis in Scottish males. It kind of looks like the north face of the Eiger, just kind of heading north and contrasts poorly with England and Wales and particularly actually with figures from Europe where they’ve passed their peak and are on the way down. And I think they found that quite alarming and made them a bit braver perhaps than they otherwise would be.*

In keeping with Kingdon’s model, the recognition of the policy problem did not automatically result in policy change or in the issue becoming a policy priority. Instead, interviewees describe a short period of time prior to the SNP election when the importance of price began to be recognised. For example, one interviewee who had been involved in developing a political party’s manifesto recalled:

*Yeah, so the manifesto development process of the political parties mostly kind of, for most of them started about a year and a half out from the elections and it was really towards the tail end of 2006 that they, that collectively the parties started to get submissions to their manifesto process around alcohol pricing controls. A little bit around minimum pricing, more around happy hour type quantity discount bans, but without much policy evidence behind it. More just on a it’s not right that something should be cheaper than water or cheaper than soft drinks, that type of thing. And the SNP were the first to pick up on that in the 2007 manifesto and at the time of writing, there were voices within other parties who were interested in it but felt it was too early to take that jump*

Following this, the advocacy group SHAAP held an expert workshop to identify potential actions to address alcohol harms. SHAAP subsequently contributed to the ‘solution’ stream by publishing the first public report calling for floor pricing in Scotland [60]. Within the expert workshop, Scottish Government representatives were involved in discussions around MUP in particular.

The ‘politics’ stream can be seen as being brought into alignment by the replacement of the Labour-Liberal Democrat Coalition by the SNP Scottish Government. According to interviewees, the former administration had expressed interest in taking action but preferred to continue a partnership approach with industry, exemplified by the establishment of the Scottish Government’s Alcohol Industry Partnership. It appears that it was not until the political environment changed following the election of the SNP that addressing alcohol price became a focus for policy. In the words of a different civil servant:

*Civil Servant: I suppose the change of administration at the government was significant in the shift of focus for alcohol policy. So, I think the old administration had recognised the problem but in terms of shifting the emphasis of what we do, it didn't happen until the new administration came in, the SNP administration came in […]*

Lastly, Kingdon’s model highlights the importance of policy entrepreneurs in facilitating policy and achieving ‘coupling of the streams’. While a number of potential policy entrepreneurs can be identified in relation to MUP, three appeared particularly prominent within the Scottish context. First, one individual (who had previously conducted a PhD on the relationship between evidence and policy [74]) who represented SHAAP was repeatedly identified as having been effective in ensuring both the problem and solution streams remained on the agenda. Second, the Chief Medical Officer had an active role in highlighting the importance of alcohol for Scotland’s public health and the potential for MUP within Scottish Government [75]. Third, the Deputy First Minister was noted to have been a key individual in helping make MUP a politically viable option by building political support within the SNP:

*Civil Servant: the really important thing to understand for somebody studying this, is that that difficulty was not at all limited to opposition parties. So the first time that, you have to understand that the proposals that were put forward around alcohol actually went forward in about, from memory, 2006/2007 and they went up to the Cabinet and, I won’t you know, quote verbatim, but the First Minister’s response at that time was reportedly far from complimentary or supportive at some of the measures that were being suggested, and the person who saved the day was actually Nicola Sturgeon, who at that time was both Health Minister and Deputy First Minister, and she persuaded the First Minister to allow her to take the issue off the table at that point, off to one side and to spend time with him talking him through the issue of alcohol on health and alcohol on society. And she was, much to her credit, able to get him to a position where he accepted almost all of the package.*

Kingdon’s multiple streams theory therefore highlights the importance of the epidemiological evidence in helping identify the policy problem. However, it was not until the development of a feasible solution by an advocacy organisation and the establishment of an amenable political context that MUP became the centre of detailed political debate. A number of individuals from a variety of backgrounds acted as policy entrepreneurs to help ‘couple’ these three streams.

#### Punctuated Equilibrium Theory

Punctuated Equilibrium Theory raises the possibility that a punctuation has occurred [23], [76]. In other words, alcohol policy may have moved from a relatively niche area of public policy that did not enjoy a high priority to become an area of broader interest to various stakeholders and susceptible to more significant policy change. Indeed, there was a general consensus among interviewees that alcohol policy had become a high-profile area that was receiving far more attention than previously. For example:

*I think it [alcohol policy] has become more of a focus for several reasons. One is I think the media find it quite a topic of interest for their readers, particularly the anti-social behaviour and binge drinking cultures. I think the objective evidence of the increasing costs of alcohol particularly in health and the rising frequency of alcoholic liver disease and alcohol related mortality, rising alcohol related admissions, I think are all, have all raised its profile and I think most of all, I think it’s been the pro-active, more pro-active approach taken by the Scottish Government.*

Consistent with Punctuated Equilibrium Theory, the role of the media in making alcohol policy a priority is emphasised by this respondent. There is therefore evidence to support the existence of a ‘punctuation’ but this raises the question of why. According to Punctuated-Equilibrium Theory, a change in a policy issue’s framing (or ‘image’) may result in an increased focus on a policy area [76].

In the case of MUP, our analysis of evidence submission documents suggests such a re-framing occurred [77]. In particular, policy advocates have worked hard to change the policy ‘image’ from tackling the misuse of alcohol, especially among young people and ‘binge drinkers’ [41], to adopting a ‘whole-population’ approach aimed at improving public health [2] that seeks to change the population distribution of alcohol consumption [78]. Interviewees’ felt that emphasising public health aspects helped redefine the policy issue in a manner conducive to MUP:

*Civil Servant: I think – and I think it was true when we did the smoking ban as well – that as soon as you talk in public health terms, it sort of, it brings the debate up to a better level. Because whenever anybody spoke to us when we were doing the smoking ban, and started talking about the impact on the business, or what might happen, you know, we would always say, “but this is about public health,” and it’s almost like public health is something which overrides anything, because how can you not do something which is in the interests of public health?*

The ‘whole-population’ policy image also highlights the importance of harms arising from, and experienced by, a far broader part of the population including impacts resulting from other’s drinking. This switch to a population-based approach was informed by the work of public-health advocates over a period of several years, including the work of international alcohol epidemiologists portraying alcohol as ‘no ordinary commodity’ [4]. Within Scotland, these ideas were captured within the influential logic model by NHS Health Scotland described above [58]. By broadening the scope of those affected adversely by alcohol, this allowed new entrants to focus on the policy issue, thus assisting in the development of new coalitions:

*Politician: So whether it’s doctors’ groups, whether it’s nursing groups, whether it’s the BMA [doctors’ trade association], you know, whoever it is – I don’t think I would single out – but it’s actually not just been health groups, it’s been like the Salvation Army for example, it’s been Children First. So it’s not just been directly health related groups. It’s been, you know, those groups who have experienced the effects that children have had and brought up in alcohol addicted households.*

Punctuated-Equilibrium Theory therefore helps identify an increased emphasis on alcohol policy that facilitated MUP’s emergence. An important contributor to the increased focus has been a re-framing of the policy ‘image’, thus encouraging an increased range of actors to participate in policy debates and facilitate the creation of broad coalitions for MUP.

#### Multi-Level Governance

While a picture of the policy process is emerging from the application of the above two theories, a number of important issues have yet to be adequately explained. These include understanding why MUP (rather than more conventional approaches) has emerged as a policy solution and why the Scottish Government has been responsible for MUP’s development rather than other UK-based institutions. It is to provide answers to these questions that we now turn to a Multi-Level Governance perspective. To do so, we will consider a number of factors that have previously been identified as potentially important in explaining policy divergence between devolved territories including the *powers* (an institution’s ability to make and implement decisions), *politics* (especially party political considerations), and the *policies* being promoted by the policy communities associated with a specific institution [79]. The smaller size of the Scottish policymaking community and a relative lack of institutional civil service capacity are thought to be associated with greater access although the importance of these factors has been questioned [80].

As noted previously, MUP was first articulated as a policy idea within Scotland by the advocacy organisation SHAAP following its organisation of an expert workshop on addressing alcohol-related harms. The Scottish Parliament’s limited powers to intervene on alcohol price were a critical factor explaining MUP’s emergence:

*Academic: From that [SHAAP] workshop, I think the proposal around minimum unit pricing emerged – largely because there was a huge body of evidence about price of alcohol, but the Scottish Government’s ability to intervene on price was obviously limited because of the tax powers lying with the UK government.*

While a consideration of the powers of the Scottish Government helps to explain the development of the *form* of intervention, it does not help explain why Scotland decided on intervention in the first place. One important explanation for a Scottish lead on alcohol policy consistently identified by interviewees was the greater burden of alcohol-related harms in Scotland than elsewhere in the UK:

*Academic: […] the other thing I think is that I mean countries don’t like to be sort of scored or measured, compared with other ones, and when you start sort of showing that one country is much worse than another country, i.e. the cirrhosis deaths in Scotland or England, I think this also makes politicians a little bit sort of embarrassed, again sort of thinking ‘oh gosh we need to do something.’*

However, while the greater burden was clearly identified as important, several other contributory factors were evident. Many respondents explained that the relatively small size of the Scottish policy community meant that access was easier for those seeking to influence policy, in keeping with previous research on the role of alcohol industries in seeking to influence Scottish alcohol policy [81]. Another important reason for Scotland developing MUP was the different political context. As noted, the Scottish Government was run by a pro-independence party and MUP was therefore seen by some respondents as a helpful way for the SNP to differentiate itself from the UK Government and articulate a distinctive Scottish approach on a major public policy issue. However, there was a policy precedent that also played an important influence on the political context. Scotland’s lead in the smoke-free public places legilsation was seen as an important facilitator in politicians and civil servants contemplating MUP:

*Civil Servant: I think it was an illustration, it provides an illustration of politicians, you know, making a public health change, which probably they wouldn't have necessarily envisaged themselves making even a year before it came in or before the proposal was announced. And it working and the sky not falling in and, you know, the public might have been against it before it came in, but certainly, you know, came round when they realised that actually it was more pleasant and, like I say, the sky didn't fall in. So, I think probably it influenced them and gave them the confidence to do something similar, knowing that actually they can make these changes, they can make a difference and, you know, public opinion, if it works, will follow them.*

The influence of the smoke-free public places legislation illustrates the importance of appreciating the longstanding and often unintended influences of previous decisions (i.e. path dependency). Previous public health policy seemed to act as a facilitator to further public health action. Similarly, the fact that alcohol licensing had already established the principle of legislative intervention within alcohol policy assisted MUP’s development. In addition, a number of interviewees expressed the view that the Scottish electorate was different from England in terms of their political support and in particular, more accepting of state intervention, a point noted in previous academic literature [38].

### Lessons for Evidence-Informed Policymaking

Our analysis of the development of MUP raises a number of potential lessons for those seeking to improve the use of evidence within public health policy. First, there is some support for those seeking evidence-informed policy [82], [83], [84]. In particular, epidemiological data was important in highlighting the problem and systematic reviews and econometric modelling appear to have played an important role in allowing those seeking policy solutions to make use of price as a mechanism to address alcohol-related harms. Also, intermediary organisations served as a ‘bridge’ between the worlds of research and policy, assisting civil servants in developing an alternative policy image that would help further a public health approach in alcohol policy [85]. However, this case study also highlights the importance of the enlightenment function of evidence [86]. In this case, the change to a population framing that emphasises alcohol as ‘no ordinary commodity’, allowed a change in how alcohol policy is conceptualised and has been crucial for MUP’s development. There therefore remains a need for synthesis, but also longer term research that results in evidence helping policymakers think about policy issues in a new way [87].

Policy entrepreneurs, responsible for helping to combine the three streams of problem, politics and policy, have played an important role in policy development and they have drawn upon evidence in varied ways to assist in this process [73]. There continues to be considerable interest in the use of knowledge brokers to help provide evidence to those responsible for decision-making and the role of NHS Health Scotland in the case of MUP provides some support for such efforts [88], [89]. However, our analysis suggests that a wide variety of other factors were at least as important and so knowledge brokers alone are unlikely to be sufficient in fostering evidence-informed policy in many situations. Furthermore, the fact that advocates, civil servants and politicians all could operate as knowledge entrepreneurs suggests that knowledge linkage efforts should perhaps operate in a broader way – linking multiple communities rather than just bridging a divide between research and policy [90].

Adopting a Multi-Level Governance perspective has highlighted a number of issues that remain under-investigated within the public health literature. MUP arguably presents an example of ‘venue shopping’ where the Scottish Government provided an additional institution for public health advocates to attempt to influence policy rather than the UK Government, raising parallels with previous tobacco control policy developments [39]. The Scottish Government did not merely act as an alternative venue but the limited institutional powers actually fostered policy innovation, with MUP being a more effective public health measure than more traditional taxation-based measures used alone [91], [92]. To capitalise on the availability of a Scottish policymaking venue, local data [56], [93] which compared Scotland unfavourably to other jurisdictions were helpful in prioritising alcohol policy. This combined with the fact that health policy is a highly visible area for Scottish Government, given its limited powers [38]. The implications of this case study arising from Multi-Level Governance do not just apply within the UK but also illustrate the potential for evidence to influence policy in other political systems with multiple levels of political representation, including North America and Europe. However, the legal challenges at the European level highlight the importance of considering how those opposed to a public health policy can seek recourse to venues more advantageous to them [81].

### Strengths and Limitations

Our study has a number of important strengths that are worth noting. We have drawn upon several sources of data to obtain a detailed understanding of a high-profile policy’s development. Furthermore, our data collection occurred in a timely fashion and we were successful in obtaining interviews with a number of actors directly involved in the policy process. Our study also makes use of a number of political science theories to better understand MUP’s development. This approach has allowed different aspects of the policy process to be understood while providing a relatively parsimonious account of the policy process.

A number of limitations should be noted. The literature on Kingdon’s multiple streams and Punctuated-Equilibrium Theory originated in the pluralist American tradition and although they have been applied to the UK setting [94], [95], debates continue about their relevance to this context. They have been critiqued for downplaying the importance of specific economic and elite interests. While in this article, with its focus on identifying potentially generalisable lessons for evidence-informed policy, we have not sought to detail the role of specific interest groups, we have begun to do so elsewhere [96], [97]. However, these two theories have been usefully applied to better understand the dynamics of the policy process and in particular allow an appreciation of the complex process by which MUP became a feasible policy solution. In addition, although our analysis in this paper has been informed by three political science theories, this has not resulted in a comprehensive account which explains all aspects of the policy process and other theories would yield additional insights. While the literature on multi-level governance has originated within Europe to help highlight the increasing diversity of policy actors and the multiple institutions involved in the policy process, an alternative body of literature on polycentric governance, points to the interplay and contestation that occurs between competing regulatory organisations, particularly given ongoing changes in institutional jurisdictions [98], [99]. This perspective could have been usefully employed for this case study, particularly to explore the latter phases of the policy’s development, including the ongoing legal challenges that have impeded MUP’s enactment. However, an adequate exploration of the contestation between institutions, including the incentive for Scottish politicians to demonstrate their leadership over the rival UK Westminster arena in the context of the Scottish independence agenda, is outside the scope of this article but we will examine this in subsequent publications [100]. Similarly, the literature on science and technology studies highlights the ambiguous nature of evidence and the processes by which research may be used strategically in policy debates [101]. For example, Latour has highlighted the malleability of evidence so that its meaning may be reinterpreted, often in strategic ways, during policy debates [102], [103]. In this case study it is clear that econometric modelling carried out by a team at Sheffield University has been particularly influential but that rival perspectives exist as to the validity and utility of this research. Again, this aspect of the policy debate will be explored in detail in subsequent analyses [104].

## Conclusion

Understanding the process by which public health policy develops holds considerable promise in improving the ability of public health practitioners and researchers to engage in policy. This article has therefore studied the development of an innovative, high-profile public health policy by taking a multiple lenses approach. The use of three perspectives to understand the policy process has provided insights which could not be attained through the use of a single theory. In addition, by building an understanding of the policy process as a whole, we have been able to appreciate the broad influences of different forms of evidence in the policy process and have been careful to avoid overemphasising the impact of evidence.

The story of MUP illustrates the complexity of the policy process and highlights the limitations of seeing policymaking as purely determined by evidence (evidence-based policy) rather than evidence as one important influence on policy [105]. While epidemiological data have served as a key indicator of a change in alcohol-related harms, epidemiological ideas have also been influential in changing thinking about the policy issue and have fostered a move to a population-based approach. In addition, evidence has been tailored to the political context so that data were presented at a politically appropriate aggregation. However, much of the MUP story does not relate to evidence but rather political and institutional factors which should not be ignored by researchers and practitioners seeking to influence the policy process.

A number of general lessons for evidence-informed policy have been suggested. We find some support for ongoing initiatives that emphasise the role of linkage between different communities and systematic reviews. However, we note the existence of multiple communities (rather than just research and policy communities) which appeared to benefit from the availability of evidence in this case study and also the importance of evidence as a source of ideas or concepts (which may not necessarily be fostered by research synthesis endeavours). More substantively, we find that institutional factors interact with evidence in complex but potentially helpful ways that not only provide increased opportunities for evidence to inform policy but may drive innovation that results in greater public health gains.
